# Causal associations between severe mental illness and sepsis: a Mendelian randomization study

**DOI:** 10.3389/fpsyt.2024.1341559

**Published:** 2024-03-12

**Authors:** Ruhao Yang, Hongyu Xiang, Ting Zheng

**Affiliations:** ^1^ Department of Emergency, Renmin Hospital of Wuhan University, Wuhan, China; ^2^ Department of Rheumatology and Immunology, Zhongnan Hospital of Wuhan University, Wuhan, China; ^3^ Department of Endocrinology, Zhongnan Hospital of Wuhan University, Wuhan, China

**Keywords:** sepsis, major depression, schizophrenia, Mendelian randomization, causal relationship

## Abstract

**Objective:**

SMI (severe mental illness) has been identified as a risk factor for sepsis in observational studies; however, the causal association between them has yet to be firmly established. We conducted MR (mendelian randomization) to unveil the causal relationship between SMI and sepsis as well as sepsis mortality.

**Methods:**

GWAS (Genome-wide association) data for major depression and schizophrenia were selected as exposure. GWAS data for sepsis and sepsis mortality were selected as outcome. Genetic variants significantly associated with the exposure (*P* value<1x10^-6^) were selected as instruments. We primarily employed the IVW (inverse-variance weighted) method for analysis. Furthermore, we employed Cochrane’s Q test to assess heterogeneity and the MR-Egger intercept test to identify horizontal pleiotropy.

**Results:**

We selected 108 SNPs (single nucleotide polymorphism) used to predict major depression and 260 SNPs that predicted schizophrenia. Genetically predicted major depression was suggestively linked to a higher sepsis risk (OR=1.13, 95%CI 1.02-1.26, *P*=0.023). In contrast, MR analysis did not find an association between schizophrenia and sepsis risk (OR=1.00, 95%CI 0.97-1.04, *P*=0.811). Furthermore, no significant causal evidence was found for genetically predicted SMI in sepsis mortality. Moreover, no heterogeneity and horizontal pleiotropy were detected.

**Conclusion:**

Our research revealed a suggestive association between genetically predicted major depression and an elevated risk of sepsis in individuals of European ancestry. This finding can serve as a reminder for clinicians to consider the possibility of subsequent infection and sepsis in depressive patients, which may help reduce the incidence of sepsis in individuals with depression.

## Introduction

Sepsis, arising from a dysregulated host response to severe infection, can result in multiple organ dysfunction (1). Sepsis has high morbidity and mortality, with approximately 1.75 million cases reported in the United States in 2014 (2), and an estimated 48.9 million cases worldwide in 2017, leading to 11 million deaths (3), making it the third leading cause of hospital deaths (4). Since sepsis imposes a huge economic burden on human health, identifying sepsis risk factors and reducing its incidence and associated mortality rates is vital. Despite deeply insights into sepsis’ pathophysiology, there remains limited knowledge about its associated risk factors.

SMI (severe mental illness) is a category of psychotic disorders that affect individual cognition, emotions, and behavioral functions, significantly impacting a person’s daily life, including social, occupational, and familial functioning. It lasts for a long time, is severe, and requires long-term professional treatment and support. SMI includes, but is not limited to, major depression, schizophrenia, and bipolar disorders. Individuals with SMI are prone to suffer from physical illness (5) such as diabetes (6) and cardiovascular disease (7). Some studies have indicated that individuals with SMI face an elevated risk of infections. Seminog OO et al. showed that SMI is a risk factor for pneumococcal disease (8). A nationwide, population-based, cross-sectional research indicated that individuals with SMI also had an elevated risk of HIV (human immunodeficiency virus), HBV (hepatitis B), and HCV (hepatitis C) (9). Furthermore, schizophrenia is associated with an increased risk of acute respiratory failure and mechanical ventilation (10). Additionally, the incidence of tuberculosis is higher in individuals with schizophrenia compared to the general population (11). A prospective population-based study showed that depression was associated with many types of infections including hepatitis, respiratory and urogenital infection (12).

Although SMI is substantiated to increase the risk of some types of infections, the association between SMI and sepsis or sepsis mortality is limited. In a prospective population-based study, Andersson NW found that depression elevated the risk of sepsis (12). The mortality in patients with SMI is significantly higher than in the general population (13). A systematic review and meta-analysis showed that patients with schizophrenia suffered an average of 14.5 years of potential life lost (14). However, when it comes to sepsis mortality, the association between SMI and sepsis mortality is controversial (15, 16). Additionally, the causal association between SMI and sepsis or sepsis mortality is restricted to traditional observational studies, in which confounding biases could not be entirely excluded. MR (mendelian randomization) can minimize confounding biases and overcome the limitations of traditional observational studies. Thus, we performed MR to better establish the causality between SMI and sepsis.

Our study aimed to investigate the causal relationships between SMI and sepsis or sepsis mortality using MR techniques. SMI includes major depression and schizophrenia. We hypothesized that there would be a directional causal effect between SMI and sepsis or sepsis mortality.

## Methods

### Study design

**Figure 1:**
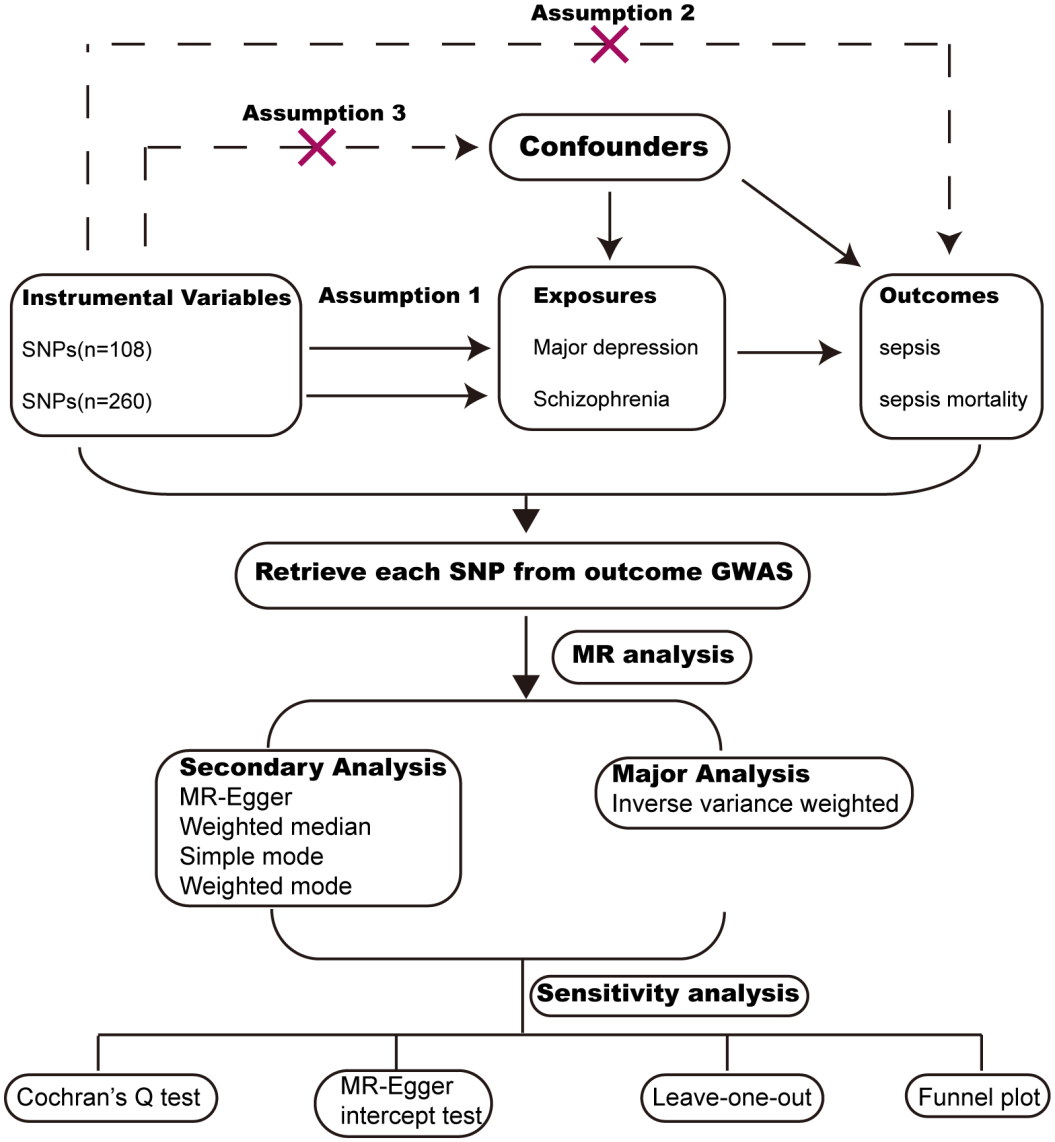
Study design chart for Mendelian randomization.

MR analysis is a widely used methodology for assessing causal relationships between exposure and outcome, with its level of evidence second only to RCTs (randomized controlled trials). We conducted MR analysis based on publicly available GWAS (Genome-wide association) data from large populations. The exposure GWAS data include major depression and schizophrenia. The outcome GWAS data include sepsis and sepsis mortality within 28 days. To reduce population bias, all the GWAS data were obtained from individuals of European ancestry (**Table 1**). MR analysis followed critical assumptions: (1) Genetic instrumental variants showed a significant association with the exposure; (2) Genetic instrumental variants have an influence on the outcome only through the exposure; (3) Genetic instrumental variants should be free from any confounding factors associated with the exposure and outcome. All original data used in this study have obtained ethical approval and informed consent from the participants. The study design chart can be found in **Figure 1**.

**Table 1:** Summary information of the data used in this study.

Trait	Data source	Year	Population	ncase	ncontrol
Exposures					
Major depression	PGC^a^	2019	European	170,756	329,443
Schizophrenia	PGC^a^	2022	European	52,017	75,889
Outcomes					
Sepsis	UK Biobank	2021	European	11,643	474,841
Sepsis mortality within 28 days	UK Biobank	2021	European	1,896	484,588

a Psychiatric Genomics Consortium.

### Data source for SMI, sepsis and sepsis mortality

The data for major depression and schizophrenia were obtained from the PGC consortium (Psychiatric Genomics Consortium). The GWAS for major depression included 500,199 participants (170,756 cases, 329,443 controls). The GWAS for schizophrenia included 127,906 participants (52,017 cases, 75,889 controls). We screened independent SNPs (single nucleotide polymorphism) with *r* ^2^<0.001 and a *P* value <1x10^-6^. The GWAS data for sepsis and sepsis mortality were obtained from the UK Biobank consortium. The IEU (Integrative Epidemiologic Unit) GWAS database (ieu-b-4980) for sepsis included 486,484 participants (11,643 cases, 474,841 controls). The IEU GWAS database (ieu-b-5086) for sepsis mortality within 28 days included 486,484 participants (1,896 cases, 484,588 controls).

### Instrumental variables selection

We screened instrumental variables to investigate causal associations between exposure and outcome. The criteria were as follows: the SNPs that have robust associations with exposures reached a genome-wide level of significance threshold (*P* value<1x10^-6^); A linkage disequilibrium threshold (*r* ^2^<0.001) and a clumping window size greater than 10,000kb were selected to avoid linkage disequilibrium; The SNPs related to the outcome (*P* value<1x10^-6^) and SNPs absent in the outcome database were excluded; Finally, We excluded SNPs with F statistics below 10 to ensure a robust association between instrumental variables and exposures. The F statistics for each SNP were calculated using the formula: F=R^2^/(1-R^2^)×(N-2), with R^2^ denoting the proportion of variance explained by the genetic instrument, and N representing the sample size in the exposures (17). The R^2^ value was calculated using formula 2×(1-MAF) MAF ×beta^2^, with MAF representing the minor allele frequency.

### MR analysis

We employed five methods to perform MR analysis, including IVW (inverse variance weighted), weighted median, MR-Egger regression, weighted mode and simple mode. Among these methods, IVW was chosen as the primary analysis. The other four methods were used as additional methods for MR analysis. A *p*-value less than 0.05 was considered nominally significant. However, for multiple testing (2 exposures × 2 outcomes = 4 tests), the level for statistical significance was adjusted to *P*=0.05/4 = 0.0125.

### Sensitivity analysis

Sensitivity analysis included assessing heterogeneity and horizontal pleiotropy. Heterogeneity among SNPs was assessed through Cochran’s Q test, with a significance level of *P*<0.05 indicating the presence of heterogeneity. Horizontal pleiotropy was calculated using the MR-Egger intercept test, with a significance level of *P*<0.05 indicating the presence of horizontal pleiotropy. Furthermore, we conducted a leave-one-out analysis to demonstrate the impact of individual SNPs on the overall estimates and employed a funnel plot to evaluate the heterogeneity.

### *Post hoc* analyses

We conducted MR analyses using sepsis as the exposure and major depression as the outcome to assess reverse causation between them. The SNPs that had strong associations with sepsis (*P* value<1x10^-5^, *r* ^2^<0.001 within 10,000kb) were selected.

### Statistical analysis

All statistical analyses in this study were performed in R (version 4.1.2) using the “TwoSampleMR” package. Significance was determined based on the Bonferroni correction, with a threshold of *P*<0.0125 (0.05/4). *P* < 0.05 was considered nominally significant. Associations that reached significance but did not survive the Bonferroni correction were regarded as suggestive associations. Causal associations were evaluated using OR (odds ratio) and 95%CI (confidence interval). Power calculations were conducted through the website http://cnsgenomics.com/shiny/mRnd/ (18).

## Results

### Characteristics of genetic instruments

After screening Genetic Instruments with a *P* value < 1x10^-6^ and *r* ^2^<0.001, we identified 108 SNPs that were used to predict major depression and 260 SNPs that predicted schizophrenia. The F statistics for these genetic instruments exceeded 10, indicating a strong relationship between exposures and outcomes. The selected SNPs are provided in **Supplementary Material**.

### MR analysis: SMI as exposure, sepsis as outcome

**Figure 2:**
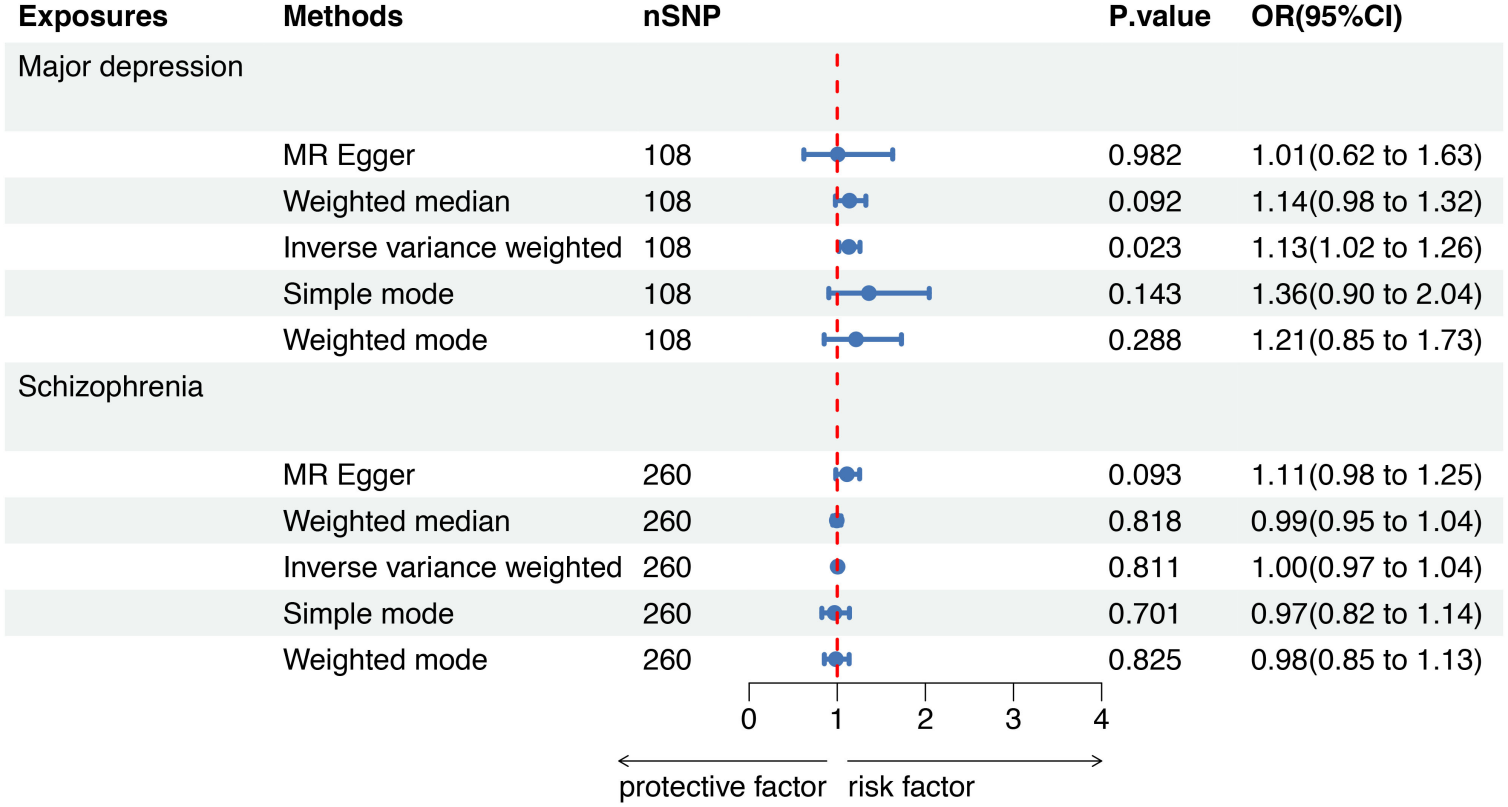
Mendelian randomization to investigate the causal impact of SMI on sepsis risk. SMI severe mental illness, SNP single nucleotide polymorphism, OR odds ratio, CI confidence interval, MR Mendelian randomization.

**Figure 3:**
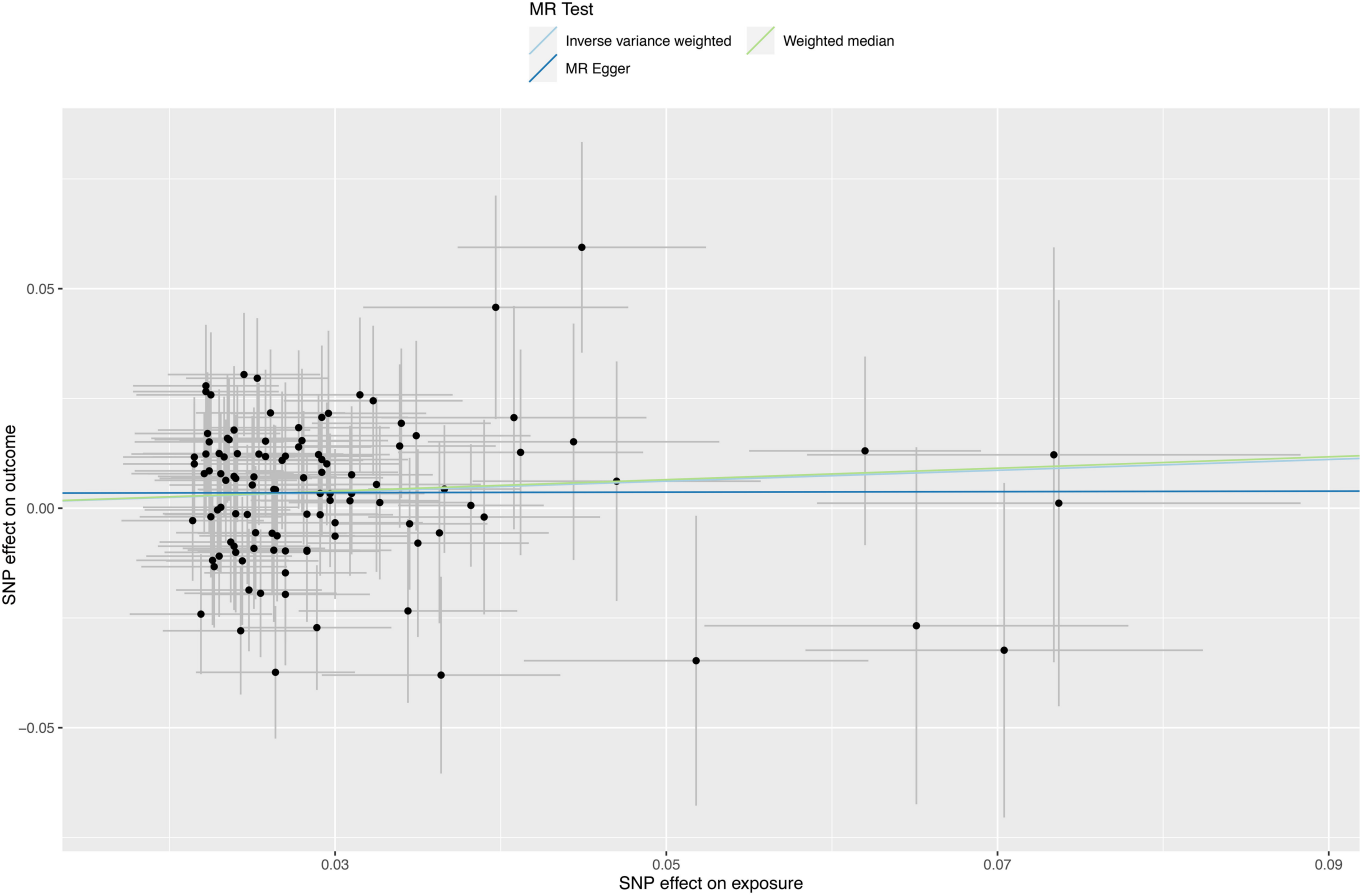
Scatter plots depicting significant estimates derived from genetically predicted major depression on sepsis. Each line’s slope signifies the causal association as per different methods. The Mendelian randomization-Egger is represented by the dark blue line, the inverse variance weighted by the blue line, and the weighted median by the green line.

A forest plot was used to demonstrate MR analysis of the causal associations between SMI and the risk of sepsis (**Figure 2**). The IVW method showed a suggestive association between major depression and sepsis (OR=1.13, 95%CI 1.02-1.26, *P*=0.023). The MR-Egger, weighted median, weighted mode, and simple mode results were directionally consistent with the IVW findings (**Figure 3**). However, no significant association was found between schizophrenia and sepsis (OR=1.00, 95%CI 0.97-1.04, *P*=0.811).

### MR analysis: SMI as exposure, sepsis mortality as outcome

**Figure 4:**
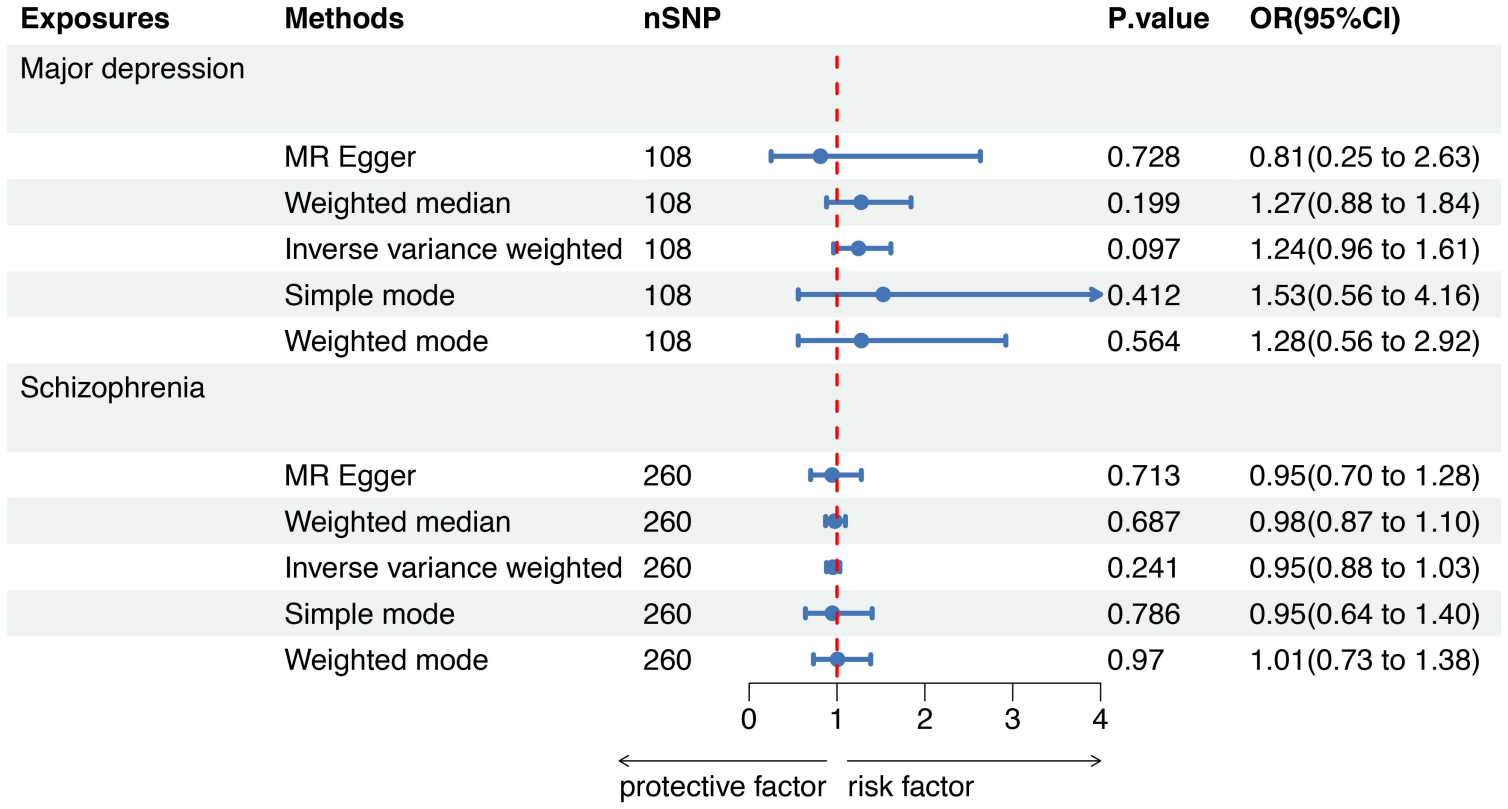
Mendelian randomization to investigate the causal impact of SMI on sepsis mortality. SMI severe mental illness, SNP single nucleotide polymorphism, OR odds ratio, CI confidence interval, MR Mendelian randomization.

To further evaluate the causal associations between SMI and the risk of sepsis mortality within 28 days, a forest plot revealed that there was no genetic causal association between major depression (OR=1.24, 95%CI 0.96-1.61, *P*=0.097) or schizophrenia (OR=0.95, 95%CI 0.88-1.03, *P*=0.241) and sepsis mortality by five MR analysis methods (**Figure 4**).

### Sensitivity analysis

**Figure 5:**
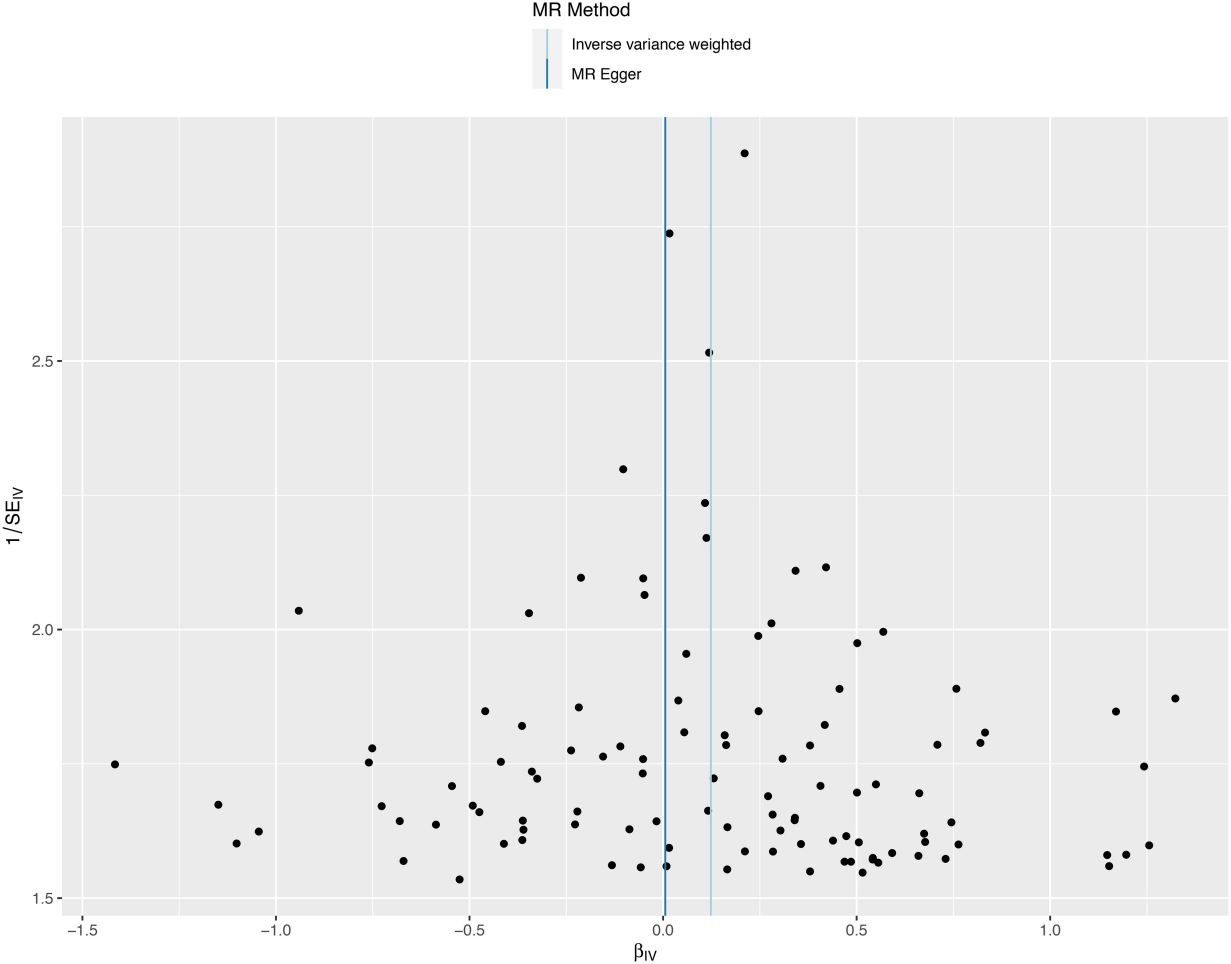
A funnel plot employed to assess heterogeneity in significant estimates arising from genetically predicted major depression on sepsis. The Mendelian randomization-Egger is depicted by the dark blue line, while the blue line represents the inverse variance weighted method.

**Figure 6:**
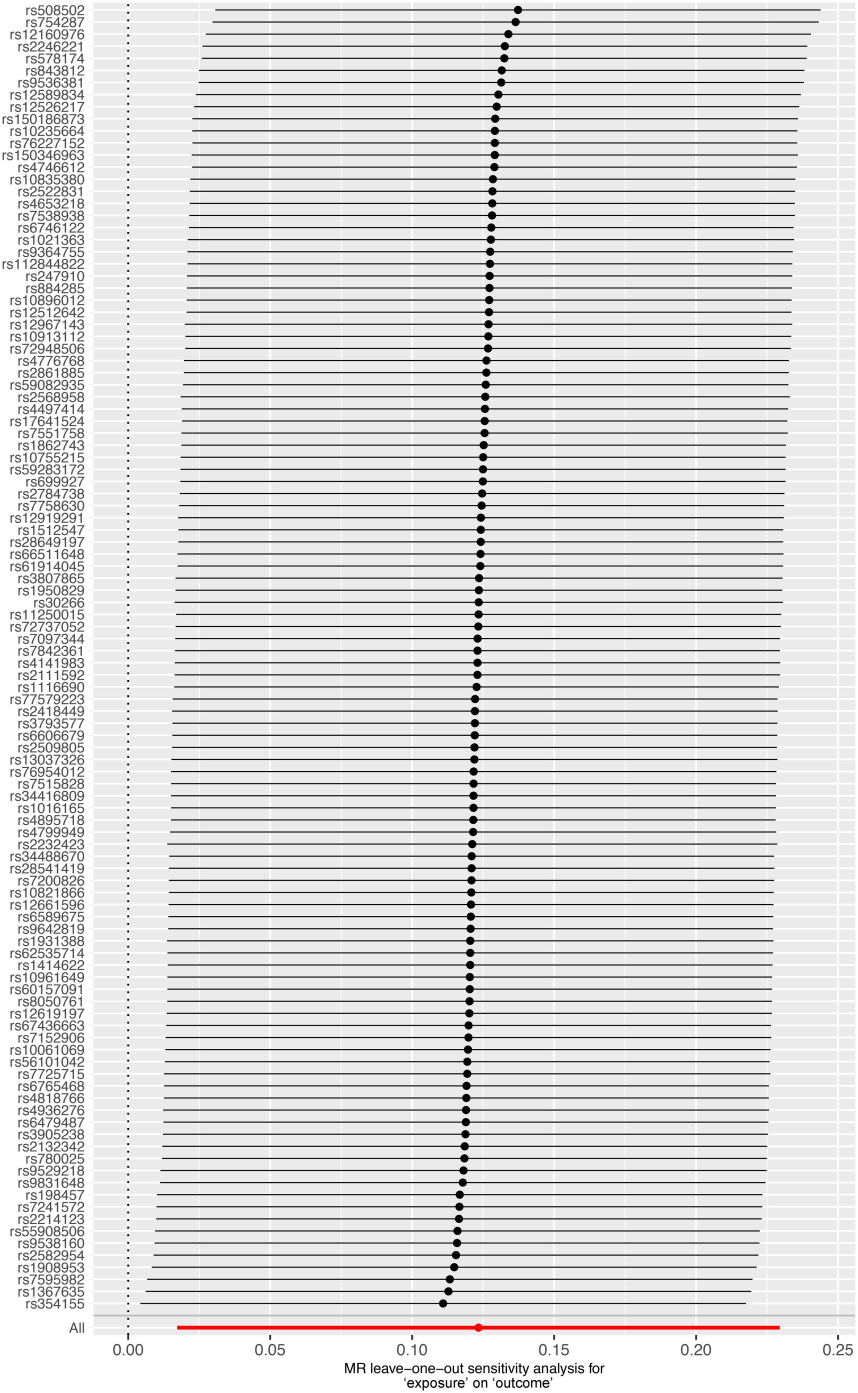
Leave-one-out plot for significant estimates derived from genetically predicted major depression on sepsis.

We conducted a sensitivity analysis to evaluate the reliability of our findings (**Table 2**). Cochran’s Q test showed no heterogeneity in the MR analysis of major depression and sepsis (Cochran’s Q=95.95, *P*=0.769), schizophrenia and sepsis (Cochran’s Q=271.87, *P*=0.279), major depression and sepsis mortality (Cochran’s Q=87.88, *P*=0.911), and schizophrenia and sepsis mortality (Cochran’s Q=276.84, *P*=0.213). Heterogeneity was visualized by a symmetrical funnel plot, showing that the data points were equally distributed around the funnel (**Figure 5**). The MR-Egger intercept test revealed the absence of horizontal pleiotropy in all analyses, with all *P*-values exceeding 0.05. Additionally, the leave-one-out analysis demonstrated that the estimates were not influenced by any individual SNP (**Figure 6**).

**Table 2:** Results of Cochrane’s Q test and pleiotropy test.

Exposures	Outcome	Cochrane’s Q test		Pleiotropy test			
		*Q*	*P value*	*Egger intercept*	*SE*	*P value*	
Major depression	Sepsis	95.95	0.769	0.003	0.007	0.625	
Schizophrenia	Sepsis	271.87	0.279	-0.006	0.004	0.094	
Major depression	Sepsis mortality	87.88	0.911	0.012	0.017	0.466	
Schizophrenia	Sepsis mortality	276.84	0.213	0.001	0.009	0.952	

### *Post hoc* analyses

Two-sample MR analysis was performed to assess the reverse causation between sepsis and major depression. We identified 16 SNPs that presented a strong association with sepsis. We observed that sepsis did not affect major depression incidence (OR 0.99, 95%CI 0.95-1.02, *P*=0.410). There was no evidence of heterogeneity (Cochran’s Q=14.75, *P*=0.469) or horizontal pleiotropy (*P*=0.128) in the MR analysis.

## Discussion

We investigated the causal connections between SMI and the risk of sepsis or sepsis mortality by MR analysis in individuals of European ancestry. Our findings suggest that genetically predicted major depression is suggestively associated with an elevated risk of sepsis, however, no evidence was found to endorse an association between schizophrenia and sepsis. Furthermore, we observed no distinct link between SMI and sepsis mortality.

Observational studies have also reported an association between major depression and sepsis. Askim et al. identified that severe depression was linked to a moderate increase in sepsis risk among the general population reporting anxiety and depression symptoms, even after controlling for BMI, lifestyle, and comorbidities factors (19). In a Danish nationwide cohort study, patients with depression exhibited a more than twofold higher risk of sepsis diagnosed according to the International Classification of Diseases (12). In a study involving community-dwelling individuals in the United States, the connection between psychological distress and an elevated long-term sepsis risk could be partially attributed to depression (20). Whereas the mechanism underlying the causal association between depression and the heightened sepsis risk remains unclear.

The change in the immune system in individuals with depression may elucidate the increased sepsis risk (19). There is evidence showing that patients with major depression have increased proinflammatory cytokines (21). Interleukin-6 (22) and CRP (C-reactive protein) (23, 24) levels are strongly increased in patients with depressive disorders. Levine J et al. reported that individuals with depression exhibited higher CSF (Cerebrospinal Fluid) levels of IL-1β (25). In addition to the innate immune response, Miller AH et al. reminded us that adaptive immune responses, especially T cell responses, also contribute to depression. The author demonstrated that reductions in the quantity and efficacy of pertinent T cell subsets might play a direct role in the onset and persistence of depression (26–28). Another research also found that an increased level of soluble interleukin-2 receptor, indicating T cell activation, in individuals with depression (29). Furthermore, the alteration of the immune system in depressed individuals is partly similar to sepsis patients. Sepsis is characterized by a destructive balance of the immune system (30). In sepsis, hyperinflammation is primarily instigated by neutrophils and cytokines, which are part of the protective innate immune (31). Anti-inflammation reactions are triggered to regulate hyperinflammation, but they often lead to sustained immunosuppression (32). Immunosuppression is marked by a reduction in immune cells, such as CD4+ and CD8+ T cells, natural killer cells, and B cells (33–35). Thus, the change in the immune system in individuals with depression could contribute to the risk of sepsis.

Conversely, some studies have demonstrated that inflammation may elevate the risk of major depression, and pro-inflammatory cytokines can influence the brain, initiating the onset of depression (36, 37). In clinical settings, psychiatric impairments were observed in some sepsis patients, even after recovery from sepsis. The main psychiatric impairments include PTSD (posttraumatic stress disorder), cognitive impairment, and depression (38). Therefore, we further explored whether reverse causality existed between them. We conducted MR analyses using major depression as the outcome and sepsis as the exposure to assess reverse causation and found no association. The observations in clinical settings were not consistent with MR analysis, which may result from differences in race and sample. In addition, some biases in observational study cannot be excluded, more comprehensive and larger studies are needed.

Schizophrenia is also an SMI. In contrast to major depression, MR analysis indicated no evidence of schizophrenia being a risk factor for sepsis. However, some observational studies have shown an association between them. Daumit GL revealed that patients with schizophrenia had a minimum of twice the adjusted odds for intensive care unit admission and death due to respiratory failure or sepsis during hospitalization (39). Tokuda Y found that sepsis (7.3%) ranked as one of the primary causes of death in acute care hospitalizations among patients with schizophrenia (40). MR results in our study were inconsistent with observational studies, which may be explained by the administration of clozapine in schizophrenic patients. Clozapine is the only medication licensed for treatment-resistant schizophrenia. Ponsford M reported that the use of clozapine substantially decreased immunoglobulin levels, potentially contributing to higher rates of pneumonia and mortality related to sepsis (41). A case report showed that a schizophrenia patient who received clozapine for 4 weeks was diagnosed with clozapine-induced pancytopenia complicated by severe sepsis (42). In the future, it may be more accurate to evaluate the causal association between schizophrenia and sepsis after eliminating the confounding factor of clozapine. For example, patients treated with clozapine could be excluded.

Regarding sepsis mortality as the outcome, there was no causal link between SMI and sepsis mortality in our study. There were few studies related to sepsis mortality in patients with SMI, and the studies showed conflicting results. Two cohort studies in Denmark reported that people with SMI (schizophrenia, unipolar depression and bipolar affective disorder) have elevated mortality in the 30 days after sepsis (15, 43). On the contrary, Oud L reported that mental illness was linked to a markedly lower risk of short-term mortality in sepsis (44). Furthermore, a nationwide, population-based cohort study indicated an enhanced outcome in septic shock for individuals with SMI compared to those without, even when accounting for social disadvantages and comorbidities in physical health (16). Thus, the association between SMI and sepsis mortality need more large clinical trials.

Our study suggests that major depression is a risk factor for sepsis incidence. This finding can serve as a reminder for clinicians to consider the possibility of subsequent infection and sepsis in depressive patients, which may help reduce the incidence of sepsis in individuals with depression. Our study results are technically credible. MR analysis is more reliable method for evaluating causal associations than traditional observational studies. To obtain a robust MR estimate, the F statistics of instrumental variables in our study exceeded 100, which is larger than the typically used value of 10. We also excluded SNPs correlated with outcomes and addressed directional pleiotropy and heterogeneity. It’s noteworthy that all participants in our study were of European ancestry, which minimized population admixture.

Nevertheless, our study is subject to several limitations. Firstly, our results are confined to individuals of European descent, the study lacks ancestral and cultural diversity. Secondly, when performing power calculations for MR analysis between major depression and sepsis (http://cnsgenom-ics.com/shiny/mRnd/), the power was calculated to be 0.68, slightly lower than the typically used value of 0.8. Thirdly, our results were based solely on publicly available GWAS data. A more comprehensive clinical investigation may lead to more compelling conclusion.

## Conclusion

Our investigation suggests that genetically predicted major depression is suggestively linked with an elevated risk of sepsis in individuals of European ancestry. This finding can serve as a reminder for clinicians to consider the possibility of subsequent infection and sepsis in depressive patients, potentially contributing to a reduction in sepsis incidence among individuals with depression.

## Data availability statement

All data are publicly available. Further inquiries can be directed to the corresponding authors.

## Author contributions

RY: Visualization, Writing – original draft. HX: Formal analysis, Writing – original draft. TZ: Writing – review & editing, Conceptualization, Writing – original draft.

## References

[1] Singer M Deutschman CS Seymour CW Shankar-Hari M Annane D Bauer M. The third international consensus definitions for sepsis and septic shock (Sepsis-3). JAMA. (2016) 315:801–10. doi: 10.1001/jama.2016.0287 PMC4968574 26903338

[2] Rhee C Dantes R Epstein L Murphy DJ Seymour CW Iwashyna TJ. Incidence and trends of sepsis in US hospitals using clinical vs claims data, 2009-2014. JAMA. (2017) 318:1241–9. doi: 10.1001/jama.2017.13836 PMC5710396 28903154

[3] Rudd KE Johnson SC Agesa KM Shackelford KA Tsoi D Kievlan DR. Global, regional, and national sepsis incidence and mortality, 1990-2017: analysis for the Global Burden of Disease Study. Lancet. (2020) 395:200–11. doi: 10.1016/S0140-6736(19)32989-7 PMC6970225 31954465

[4] Sevransky JE Rothman RE Hager DN Bernard GR Brown SM Buchman TG. Effect of vitamin C, thiamine, and hydrocortisone on ventilator- and vasopressor-free days in patients with sepsis: the VICTAS randomized clinical trial. JAMA. (2021) 325:742–50. doi: 10.1001/jama.2020.24505 PMC7903252 33620405

[5] DEH M Correll CU Bobes J Cetkovich-Bakmas M Cohen D Asai I. Physical illness in patients with severe mental disorders. I. Prevalence, impact of medications and disparities in health care. World Psychiatry. (2011) 10:52–77. doi: 10.1002/j.2051-5545.2011.tb00014.x 21379357 PMC3048500

[6] Ribe AR Laursen TM Sandbaek A Charles M Nordentoft M Vestergaard M. Long-term mortality of persons with severe mental illness and diabetes: a population-based cohort study in Denmark. Psychol Med. (2014) 44:3097–107. doi: 10.1017/S0033291714000634 25065292

[7] Laursen TM Wahlbeck K Hallgren J Westman J Osby U Alinaghizadeh H. Life expectancy and death by diseases of the circulatory system in patients with bipolar disorder or schizophrenia in the Nordic countries. PloS One. (2013) 8:e67133. doi: 10.1371/journal.pone.0067133 23826212 PMC3691116

[8] Seminog OO Goldacre MJ. Risk of pneumonia and pneumococcal disease in people with severe mental illness: English record linkage studies. Thorax. (2013) 68:171–6. doi: 10.1136/thoraxjnl-2012-202480 23242947

[9] Bauer-Staeb C Jorgensen L Lewis G Dalman C Osborn DPJ Hayes JF. Prevalence and risk factors for HIV, hepatitis B, and hepatitis C in people with severe mental illness: a total population study of Sweden. Lancet Psychiatry. (2017) 4:685–93. doi: 10.1016/S2215-0366(17)30253-5 PMC5573766 28687481

[10] Chen YH Lin HC Lin HC. Poor clinical outcomes among pneumonia patients with schizophrenia. Schizophr Bull. (2011) 37:1088–94. doi: 10.1093/schbul/sbq019 PMC3160214 20339152

[11] Ohta Y Nakane Y Mine M Nakama I Michitsuji S Araki K. The epidemiological study of physical morbidity in schizophrenics–2. Association between schizophrenia and incidence of tuberculosis. Jpn J Psychiatry Neurol. (1988) 42:41–7. doi: 10.1111/j.1440-1819.1988.tb01954.x 3260975

[12] Andersson NW Goodwin RD Okkels N Gustafsson LN Taha F Cole SW. Depression and the risk of severe infections: prospective analyses on a nationwide representative sample. Int J Epidemiol. (2016) 45:131–9. doi: 10.1093/ije/dyv333 26708840

[13] de Mooij LD Kikkert M Theunissen J Beekman ATF de Haan L Duurkoop P. Dying too soon: excess mortality in severe mental illness. Front Psychiatry. (2019) 10:855. doi: 10.3389/fpsyt.2019.00855 31920734 PMC6918821

[14] Hjorthoj C Sturup AE McGrath JJ Nordentoft M. Years of potential life lost and life expectancy in schizophrenia: a systematic review and meta-analysis. Lancet Psychiatry. (2017) 4:295–301. doi: 10.1016/S2215-0366(17)30078-0 28237639

[15] Ribe AR Vestergaard M Katon W Charles M Benros ME Vanderlip E. Thirty-day mortality after infection among persons with severe mental illness: A population-based cohort study in Denmark. Am J Psychiatry. (2015) 172:776–83. doi: 10.1176/appi.ajp.2015.14091100 25698437

[16] Lakbar I Leone M Pauly V Orleans V Srougbo KJ Diao S. Association of severe mental illness and septic shock case fatality rate in patients admitted to the intensive care unit: A national population-based cohort study. PloS Med. (2023) 20:e1004202. doi: 10.1371/journal.pmed.1004202 36913434 PMC10042353

[17] Flatby HM Ravi A Damas JK Solligard E Rogne T. Circulating levels of micronutrients and risk of infections: a Mendelian randomization study. BMC Med. (2023) 21:84. doi: 10.1186/s12916-023-02780-3 36882828 PMC9993583

[18] Brion MJ Shakhbazov K Visscher PM. Calculating statistical power in Mendelian randomization studies. Int J Epidemiol. (2013) 42:1497–501. doi: 10.1093/ije/dyt179 PMC3807619 24159078

[19] Askim A Gustad LT Paulsen J Reitan SK Mehl A Mohus RM. Anxiety and depression symptoms in a general population and future risk of bloodstream infection: the HUNT study. Psychosom Med. (2018) 80:673–9. doi: 10.1097/PSY.0000000000000619 29923889

[20] Ojard C Donnelly JP Safford MM Griffin R Wang HE. Psychosocial stress as a risk factor for sepsis: a population-based cohort study. Psychosom Med. (2015) 77:93–100. doi: 10.1097/PSY.0000000000000120 25469683 PMC4293326

[21] Alesci S Martinez PE Kelkar S Ilias I Ronsaville DS Listwak SJ. Major depression is associated with significant diurnal elevations in plasma interleukin-6 levels, a shift of its circadian rhythm, and loss of physiological complexity in its secretion: clinical implications. J Clin Endocrinol Metab. (2005) 90:2522–30. doi: 10.1210/jc.2004-1667 15705924

[22] Tiemeier H Hofman A van Tuijl HR Kiliaan AJ Meijer J Breteler MM. Inflammatory proteins and depression in the elderly. Epidemiology. (2003) 14:103–7. doi: 10.1097/00001648-200301000-00025 12500057

[23] Ford DE Erlinger TP. Depression and C-reactive protein in US adults: data from the Third National Health and Nutrition Examination Survey. Arch Intern Med. (2004) 164:1010–4. doi: 10.1001/archinte.164.9.1010 15136311

[24] Danner M Kasl SV Abramson JL Vaccarino V. Association between depression and elevated C-reactive protein. Psychosom Med. (2003) 65:347–56. doi: 10.1097/01.PSY.0000041542.29808.01 12764206

[25] Levine J Barak Y Chengappa KN Rapoport A Rebey M Barak V. Cerebrospinal cytokine levels in patients with acute depression. Neuropsychobiology. (1999) 40:171–6. doi: 10.1159/000026615 10559698

[26] Miller AH. Depression and immunity: a role for T cells? Brain Behav Immun. (2010) 24:1–8. doi: 10.1016/j.bbi.2009.09.009 19818725 PMC2787959

[27] Irwin MR Miller AH. Depressive disorders and immunity: 20 years of progress and discovery. Brain Behav Immun. (2007) 21:374–83. doi: 10.1016/j.bbi.2007.01.010 17360153

[28] Zorrilla EP Luborsky L McKay JR Rosenthal R Houldin A Tax A. The relationship of depression and stressors to immunological assays: a meta-analytic review. Brain Behav Immun. (2001) 15:199–226. doi: 10.1006/brbi.2000.0597 11566046

[29] Mossner R Mikova O Koutsilieri E Saoud M Ehlis AC Muller N. Consensus paper of the WFSBP Task Force on Biological Markers: biological markers in depression. World J Biol Psychiatry. (2007) 8:141–74. doi: 10.1080/15622970701263303 17654407

[30] van der Poll T Shankar-Hari M Wiersinga WJ. The immunology of sepsis. Immunity. (2021) 54:2450–64. doi: 10.1016/j.immuni.2021.10.012 34758337

[31] Wiersinga WJ Leopold SJ Cranendonk DR van der Poll T. Host innate immune responses to sepsis. Virulence. (2014) 5:36–44. doi: 10.4161/viru.25436 23774844 PMC3916381

[32] Torres LK Pickkers P van der Poll T. Sepsis-induced immunosuppression. Annu Rev Physiol. (2022) 84:157–81. doi: 10.1146/annurev-physiol-061121-040214 34705481

[33] Hotchkiss RS Tinsley KW Swanson PE Schmieg RE Jr. Hui JJ Chang KC. Sepsis-induced apoptosis causes progressive profound depletion of B and CD4+ T lymphocytes in humans. J Immunol. (2001) 166:6952–63. doi: 10.4049/jimmunol.166.11.6952 11359857

[34] Jensen IJ Sjaastad FV Griffith TS Badovinac VP. Sepsis-induced T cell immunoparalysis: the ins and outs of impaired T cell immunity. J Immunol. (2018) 200:1543–53. doi: 10.4049/jimmunol.1701618 PMC5826615 29463691

[35] Chang K Svabek C Vazquez-Guillamet C Sato B Rasche D Wilson S. Targeting the programmed cell death 1: programmed cell death ligand 1 pathway reverses T cell exhaustion in patients with sepsis. Crit Care. (2014) 18:R3. doi: 10.1186/cc13176 24387680 PMC4056005

[36] Dantzer R O’Connor JC Freund GG Johnson RW Kelley KW. From inflammation to sickness and depression: when the immune system subjugates the brain. Nat Rev Neurosci. (2008) 9:46–56. doi: 10.1038/nrn2297 18073775 PMC2919277

[37] Leonard B Maes M. Mechanistic explanations how cell-mediated immune activation, inflammation and oxidative and nitrosative stress pathways and their sequels and concomitants play a role in the pathophysiology of unipolar depression. Neurosci Biobehav Rev. (2012) 36:764–85. doi: 10.1016/j.neubiorev.2011.12.005 22197082

[38] Desai SV Law TJ Needham DM. Long-term complications of critical care. Crit Care Med. (2011) 39:371–9. doi: 10.1097/CCM.0b013e3181fd66e5 20959786

[39] Daumit GL Pronovost PJ Anthony CB Guallar E Steinwachs DM Ford DE. Adverse events during medical and surgical hospitalizations for persons with schizophrenia. Arch Gen Psychiatry. (2006) 63:267–72. doi: 10.1001/archpsyc.63.3.267 16520431

[40] Tokuda Y Obara H Nakazato N Stein GH. Acute care hospital mortality of schizophrenic patients. J Hosp Med. (2008) 3:110–6. doi: 10.1002/jhm.256 18438807

[41] Ponsford M Castle D Tahir T Robinson R Wade W Steven R. Clozapine is associated with secondary antibody deficiency. Br J Psychiatry. (2018) 214:1–7. doi: 10.1192/bjp.2018.152 30259827 PMC6429246

[42] Pushpakumara J Karunarathna P Sivathiran S Liyanage A Indrakumar J. Clozapine induced pancytopenia leading to severe sepsis: an unusual early complication. BMC Res Notes. (2015) 8:792. doi: 10.1186/s13104-015-1777-5 26674072 PMC4681016

[43] Davydow DS Ribe AR Pedersen HS Vestergaard M Fenger-Gron M. The association of unipolar depression with thirty-day mortality after hospitalization for infection: A population-based cohort study in Denmark. J Psychosom Res. (2016) 89:32–8. doi: 10.1016/j.jpsychores.2016.08.006 27663108

[44] Oud L Garza J. Impact of history of mental disorders on short-term mortality among hospitalized patients with sepsis: A population-based cohort study. PloS One. (2022) 17:e0265240. doi: 10.1371/journal.pone.0265240 35271683 PMC8912146

