# Supplementary material for: Causal associations between severe mental illness and sepsis: a Mendelian randomization study
Source: Front Psychiatry. 2024 Mar 12;15:1341559. doi: 10.3389/fpsyt.2024.1341559 (PMC10964346; doi:10.3389/fpsyt.2024.1341559)
Supplement: Supplementary file 3 [file DataSheet_3.docx]

In medical research, one of the primary objectives is to identify exposures. Exposures are often investigated in observational studies since randomized controlled trials (RCTs) cannot always be conducted due to their excessive cost and impracticality. However, an observed exposure-outcome association may not reflect a causal relationship but may arise as a result of confounding or reverse causation. Consequently, observational studies often fail to provide unbiased estimates of the true association between exposure and outcome.

To address this issue, The instrumental variable method was proposed as an alternative statistical approach for investigating the causal relationship between exposure-outcome associations while accounting for confounding factors. In medical research, genetic variants, such as single-nucleotide polymorphisms (SNPs), serve as instrumental variables. During human gamete formation, the alleles of a given SNP are randomly assigned to egg or sperm cells before any exposure or outcome occurs. These genetic instruments are unmodifiable, ensuring lifelong exposure and alleviating concerns about reverse causation. Additionally, they remain unaffected by potentially confounding environmental exposures.

In Mendelian randomization (MR) analysis, genetic instruments must adhere to critical assumptions: (1) Genetic instrumental variants must show a significant association with the exposure; (2) Genetic instrumental variants must influence the outcome only through the exposure; (3) Genetic instrumental variants should be free from any confounding factors associated with the exposure and outcome.

The term "Mendelian randomization" (MR) was coined due to its relation to Mendel’s Laws. Randomization occurs when the alleles of a given SNP are randomly allocated to egg/sperm cells, ensuring the comparability of SNPs with respect to any known and unknown confounders. As a result, the effect of the exposure on the outcome can be unbiasedly estimated, allowing for the assessment of causality in observed associations.
